# Sedentary Behaviour and Physical Activity of People with Stroke in Rehabilitation Hospitals

**DOI:** 10.1155/2014/591897

**Published:** 2014-03-19

**Authors:** Anna Sjöholm, Monica Skarin, Leonid Churilov, Michael Nilsson, Julie Bernhardt, Thomas Lindén

**Affiliations:** ^1^Department of Clinical Neuroscience and Rehabilitation, Institute of Neuroscience and Physiology, The Sahlgrenska Academy, Gothenburg University, Gothenburg, Sweden; ^2^Department of Florey, University of Melbourne, Melbourne, Australia; ^3^Hunter Medical Research Institute, Newcastle, Australia; ^4^La Trobe University, Melbourne, Australia; ^5^Stroke Division, Florey Institute of Neuroscience and Mental Health, Melbourne, Australia

## Abstract

*Background*. Sedentary behaviour is associated with health risks, independent of physical activity. This study aimed to investigate patterns of sedentary behaviour and physical activity among stroke survivors in rehabilitation hospitals. *Methods*. Stroke survivors admitted to four Swedish hospital-based rehabilitation units were recruited ≥7 days since stroke onset and their activity was measured using behavioural mapping. Sedentary behaviour was defined as lying down or sitting supported. *Results*. 104 patients were observed (53% men). Participants spent an average of 74% (standard deviation, SD 21%) of the observed day in sedentary activities. Continuous sedentary bouts of ≥1 hour represented 44% (SD 32%) of the observed day. A higher proportion (30%, SD 7%) of participants were physically active between 9:00 AM and 12:30 PM, compared to the rest of the observed day (23%, SD 6%, *P* < 0.0005). Patients had higher odds of being physically active in the hall (odds ratio, OR 1.7, *P* = 0.001) than in the therapy area. *Conclusions*. The time stroke survivors spend in stroke rehabilitation units may not be used in the most efficient way to promote maximal recovery. Interventions to promote reduced sedentary time could help improve outcome and these should be tested in clinical trials.

## 1. Introduction

Sedentary behaviour activities with an energy expenditure of ≤1.5 metabolic equivalent units [1, 2], such as lying down or sitting) [3] is associated with a variety of health risks, regardless of physical activity levels and other traditional risk factors such as smoking and high blood pressure. Cardiovascular disease [4, 5], type 2 diabetes [6], metabolic syndrome [7], and breast cancer [8] are a few diseases connected to sedentary behaviour, which further increases all-cause mortality [4, 5, 9, 10]. Each additional hour spent sedentary has been proposed to be associated with a progressive rise in mortality risk [4]. It is, however, not only the total amount of sedentary time which seems to be important, but also the way in which it is accumulated. Prolonged bouts of time in sedentary activities (such as sitting down) have been shown to be particularly harmful, with relative benefits noted from regular brief periods of standing or walking [11].

While it is well established that patients in stroke rehabilitation are very inactive [12], sedentary behaviour, per se, is not commonly investigated [13]. In the hospital setting, a recent review [12] of 24 studies demonstrated that stroke survivors on average are involved in nontherapeutic or low physical activity for as much as 76% of the day. Few of these studies, however, reported on sedentary behaviours, that is, time sitting or lying down. In a Norwegian acute stroke unit sedentary time accounted for 77% of the day [14, 15], while the same number for Australian acute stroke units was 88% [14]. In community settings, another recent systematic review [13] found that in the few studies where sedentary time was reported, it was estimated to be between 63% and 87% of the day.

Moreover, how sedentary time or physical activity is distributed over the day in hospital settings has rarely been examined. An American report from 1980 exploring patterns of time spent with a therapist in a stroke rehabilitation unit indicates large variations throughout the day. However, overall time spent sedentary or physically active was not considered [16]. Another study conducted in Australia found that 59% of physical activity in patients admitted to acute stroke units occurred between 9:00 AM and 12:30 PM, while rest periods were more commonly enforced in the afternoon [17]. Stroke survivors in rehabilitation centres in Switzerland and Belgium were in one study reported to spend more time in the therapy areas in the morning (9:10–11:50 AM) and early afternoon (1:15–3:15 PM), although not necessarily indicating the highest levels of physical activity taking place between these hours [18].

Research indicates that the perceived and objective attributes of the environment can profoundly affect physical activity [19, 20]. Which locations within the clinics help to promote stroke survivors to be more physically active may therefore be important knowledge in order to facilitate additional activity and avoid extended bouts of sedentary behaviour. Current data in this field are limited. Although several reports describe where patients in stroke rehabilitation spend their time [12], only one study presents where activity is most likely to occur [21]. In this small study, patients were most active in the therapy area (45% of time was spent active), while they were least active in the bedroom (12%) which was where they spent a clear majority of their day (74% on weekdays and 89% during the weekend).

In this secondary analysis of data from a multisite observational study [22], our primary aim was to evaluate stroke survivors' activity patterns in Swedish stroke rehabilitation clinics to determine (1) how much sedentary time patients are involved in during the day and (2) the pattern in which sedentary time is accumulated (short versus long bouts). Our secondary aim was to examine (3) patterns of physical activity, including variation in physical activity across the day and (4) in what locations within the rehabilitation units stroke survivors are more likely to be active.

## 2. Methods

### 2.1. Study Setting and Population

The study was conducted in four different rehabilitation clinics in South-Western Sweden: SÄS/Borås Hospital, NU/Uddevalla Hospital, Sahlgrenska University Hospital, and SKAS/Skövde Hospital. The four centres all specialise in stroke rehabilitation and have an emphasis on multidisciplinary care. While three of the four units are comprehensive, combining acute, and rehabilitative care, one is exclusively a rehabilitation unit. Patients with a definite diagnosis of stroke, as defined by the World Health Organization [23], admitted to one of the four units were eligible for recruitment. Further inclusion criteria were ≥7 days since stroke onset and ≥18 years of age. Patients under palliative care were excluded from the study. Exclusion also applied to those unable to give informed consent due to cognitive impairment and if they had no relatives to consent on their behalf. The study was approved by the Regional Ethical Review Board of Gothenburg, registration number 421-09.

### 2.2. Observational Technique

Data collection consisted of behavioural mapping, based on a technique reported as both valid [12] and reliable [23]. Every 10 minutes (except for 1 randomly selected 30-minute break between 11:20 AM and 1:40 PM) during 1 weekday from 8:00 AM to 5:00 PM a trained rater observed each patient for 1 minute. This resulted in a total of 51 possible observations per patient or 54 in those cases where another trained rater was available to step in during the break. The order in which patients were observed was kept constant. At each time point, the patient's activity (e.g., sitting, walking), location (e.g., bedroom, therapy area), and company (e.g., nurse, family) were recorded using a standardized protocol. In the case of patients moving between different locations during a single observation, the place in which they spent more time was registered. Patients were also followed offward if feasible within the time limit of the next scheduled observation (e.g., in the stairwell, around the hospital entrance, in nearby outdoor areas, and in the cafeteria). No observations were performed in private situations, such as when patients visited the bathroom with closed door. Activity during unobserved periods was determined by questioning either the patient or the caregiver.

### 2.3. Demographic Data

Demographic variables (gender, age, time since stroke, side of lesion, living arrangements, and premorbid mobility) were collected from the medical records. Stroke subtype was determined according to the Oxfordshire Community Stroke Project classification [24]. Stroke severity at admission to hospital was recorded using the National Institutes of Health Stroke Scale [25, 26] and premorbid disability was classified through Modified Rankin Scale [27]. Information about patients' motor function status on the day of observation was established by means of the Mobility Scale for Acute Stroke [28]. Activities of daily living were extracted from the medical records or determined by a local nurse or an occupational therapist according to the Barthel Index [29].

### 2.4. Procedure

Before initiating data collection, the rehabilitation staff were informed that patient activity would be recorded. Patients identified as eligible were verbally and in writing informed about the practical details of the study and that it was part of the process to develop the treatment methods for training after stroke. They were also informed that participation in the study was voluntary and withdrawal is possible at any time without stating a reason. Once patients expressed their willingness to participate, informed consent was documented by their signature.

The importance of everyone in the ward trying to ignore the observer and not to change any procedures or behaviours was strongly emphasized. Observations were made on randomly selected weekdays until 104 patients had been monitored. Before each observation day, sites were screened to determine whether observation was feasible at that time. A minimum of 2 patients who met inclusion criteria and provided informed consent was required to make observation feasible. In case of more than 7 patients available per observer, the 7 with earliest birth dates in any month were to be recruited to prevent selection bias.

### 2.5. Data Processing and Categorization of Activity

Data were scanned from the forms to a computer and downloaded to a secure database at the National Stroke Research Institute, Melbourne, Australia, via remote access. All information is stored within the regulations for management and archiving of research documents at the University of Gothenburg. The database (Microsoft Access 2000) was designed to automatically register the highest level of physical activity at every 10-minute interval. Activities were grouped into 4 different categories (see Table 1) previously judged by an expert group to reflect the degree of physical effort required. In this study, time spent in categories 1 (nil physical activity, i.e., lying in bed) and 2 (minimal physical activity, i.e., sitting supported) was considered as sedentary in accordance with the definition of Pate et al. [3] that “Sedentary behaviour refers to activities that do not increase the energy expenditure substantially above the resting level and includes activities such as sleeping, sitting, lying down, and watching television, and other forms of screen-based entertainment; or those activities that involve energy expenditure at the level of 1.0–1.5 metabolic equivalent units (METs).” One MET has further been defined as “the energy expended while sitting quietly and is approximately equal to 3.5 mL of oxygen uptake per kilogram of body weight per minute for a 70 kg adult” [1, 2]. Patients in this study were classed as “active” or “physically active” when spending time in categories 3 (light physical activity, e.g., sitting unsupported) and 4 (moderate physical activity, e.g., standing, walking) [2].

### 2.6. Statistical Analyses

Statistical analyses were conducted using SPSS version 20.0 and Stata v12IC. Interrater reliability was established by two trained raters independently recording the same patients for a total of 112 observations. Agreement between the observers was tested using Cohen's Kappa, with the Kappa coefficients calculated separately for activity category, location, and people present, respectively.

Data were pooled across clinics and over days. Time spent in each activity category was estimated from the frequency of occurrence in observations, presuming that activity within every 1 minute of observation was representative for the following 9 unobserved minutes. Sedentary behaviour was calculated in relative numbers for each patient as a function of time, 100% representing a full day ranging from 8:00 AM to 5:00 PM (missing data excluded), and analysis included only data where the patient was directly observed.

To address our primary aim of examining the amount of sedentary behaviour and how it is accumulated (aims one and two), we calculated the average duration and number of bouts of sedentary behaviours across the observed day. The start and the end of a bout were characterized by a transition from an activity in AC 1 or 2 to an activity in category AC 3 or 4. To meet our secondary aim of examining daily variations in patterns of physical activity (aim three), we estimated the proportion of patients involved in light or moderate physical activity at each observation and grouped this into “time from 9:00 AM to 12:30” (as this period previously has been shown to encompass the most active part of a patients day [17]) versus “remaining time.” Independent samples Student's *t*-test was then used to test statistical significance between the groups. To test our secondary aim regarding in which locations patients were more likely to be active (aim four), activity levels were dichotomized into physically active (activity categories 3 and 4) and sedentary (activity categories 1 and 2). Two separate multilevel random-effect logistic regression models with the dichotomized activity being an output and longitudinal observations nested within individual patients (level variable) were then performed to estimate the odds of patients being physically active in all locations as compared to the bedroom (model 1) and to the therapy area (model 2), where patients in a previous study [21] were found to be least (bedroon) and most (therapy area) physically active.

## 3. Results

### 3.1. Settings

During the 10-month period of data collection (November 2009 to September 2010) each clinic had between 17 and 21 beds dedicated to stroke patients. Staff-to-patient ratios during weekdays across the clinics ranged from: 1 : 2 to 1 : 3 for nurses (including nurse assistants), 1 : 4 to 1 : 8 for physiotherapists, 1 : 4 to 1 : 8 for occupational therapists, 1 : 14 to 1 : 90 for speech therapists, and 1 : 5 to 1 : 7 for physicians. Therapists generally worked from 7:30–8:00 AM until 4:00-5:00 PM and only during weekdays. All units had comprehensive therapy departments separate from the ward. Three of the units also had a therapy room on the ward. Breakfast was normally served at 8:00–8:30 AM, lunch between 11:30 AM and 12:30 PM, afternoon snack at 2:30–3:00 PM, and dinner 4:30–5:00 PM. One of the clinics had general rest periods at 9:00-10:00 AM, 3:30–4:30 PM, and 6:00-7:00 PM. Another clinic had resting time at 1:00–1:30 PM and some patients in the remaining two centres had individually timetabled rest on their personal daily schedule.

### 3.2. Participants

Of the stroke survivors who were invited to participate, 90% (*n* = 104) were recruited. Reasons for nonparticipation included unexpected discharge before the observation day (3%, *n* = 3), isolation due to infection (1%, *n* = 1), deterioration to palliative care status (1%, *n* = 1), and refusal to participate (6%, *n* = 6). The number of participants was equally divided between the hospitals and no one was restricted to bed rest on the day of observation. Fifty-three percent (*n* = 55) of participants were male and the mean age was 70.3 years (standard deviation, SD 14.4 years, range 34–93). Time since stroke had a median of 19 days (interquartile range, IQR, reported as the 25th and 75th percentiles, 12–34 days, range 7–142), the median of the Barthel Index score was 65 points (IQR 35–95 points, range 0–100), and Mobility Scale for Acute Stroke had a median of 28 points (IQR 16–30, range 5–30). Participant characteristics for this sample have been previously reported [22].

### 3.3. Interrater Reliability and Observations

Agreement between the raters was found to be very high, with a Kappa coefficient of 0.98 for activity category, 0.99 for location, and 0.86 for company (*P* < 0.0005). In total 5376 observations were performed. On 6% of these occasions (*n* = 338) patient activity was not ascertained, mainly, while patients were in the bathroom (2%, *n* = 98), offward (2%, *n* = 89), or had speech therapy behind closed doors (1%, *n* = 62). Location was missing in only 6 observations (0.1%) altogether.

### 3.4. Sedentary Behaviour

Patients were engaged in sedentary behaviour for 74% (SD 21%) or 6.2 hours per observed day. Twenty-eight percent (*n* = 29) of patients spent >90% of the day sedentary and 19% (*n* = 20) were never observed standing or walking. The average duration of bouts of sedentary time between 8:00 AM and 5:00 PM was 38 minutes (SD 44%) but this ranged from 10 to 490 minutes. The mean number of sedentary bouts per patient per day was 9.9. While 8% of the day was dedicated to very short sedentary bouts of one single observation, on average 54% (SD 30%) of the day was spent in bouts of sedentary behaviours of more than half an hour duration. Sedentary bouts lasting for 1 hour or longer accounted for 44% (SD 32%) of the day. Participants' longest uninterrupted sedentary bout per day was on average 1.93 hours (SD 1.2 hours). Light or moderate physical activity overall accounted for 25% (SD 19%) of the time, with 13% of the observed day spent standing or walking.

### 3.5. Variation in Physical Activity across the Day

**Figure 1:**
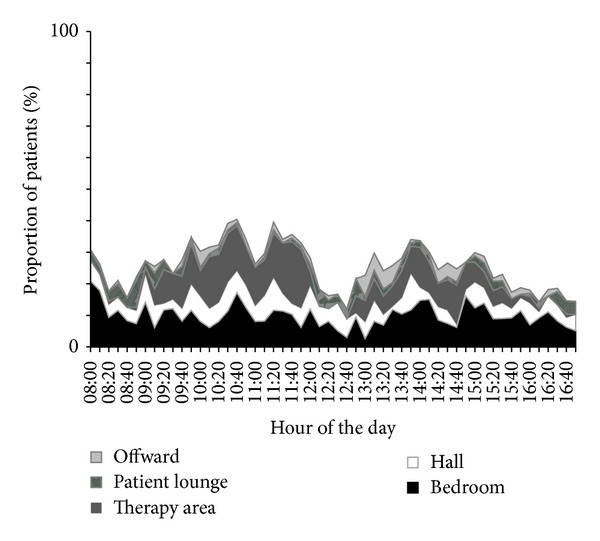
Proportion of patients (*n* = 104) engaged in light or moderate physical activity and their location at each time point.

On average 26% of patients were lightly or moderately physically active at each time point (see Figure 1), but physical activity levels varied between 12% and 40% of patients active at each observation point throughout the observed day. Few patients (16–21%) were active around breakfast time at 08:20–08:40 AM. A higher proportion (30%, SD 7%) of patients were engaged in light or moderate physical activity between 9:00 AM and 12:30 PM, compared to the rest of the observed day (23%, SD 6%, *P* < 0.0005). However, a decrease in physical activity was seen already at 12:10, with less patients (12–18%) being physically active during lunch time (12:10–12:40 PM). After 15:30 PM, activity for many again decreased (14–19% of patients active).

### 3.6. Locations Where Patients Were More Physically Active

Physical activity also varied depending on patient location, which accounted for 63% of the overall variability taking into consideration the longitudinal nature of the data across the day. In comparison to the therapy area, patients had higher odds of being physically active in the hall (OR 1.7, *P* = 0.001, confidence interval, CI 1.2–2.4). They had about the same odds of being active offward (e.g., in the stairwell, outdoors, and in the cafeteria) as in the therapy area (OR 1.1, *P* = 0.795, CI 0.7–1.7), while having lower odds in all remaining locations (OR 0.1-0.2, *P* < 0.0005, CI 0.1–0.4). When compared to the bedroom, the odds of being physically active were lower in the lounge (OR 0.4, *P* < 0.0005, CI 0.3–0.5) and about the same in the bathroom (OR 1.1, *P* = 0.788, CI 0.5–2.4). Compared to the bedroom, patients had higher odds of being active in the therapy area (OR 5.4, *P* < 0.0005, CI 4.3–6.9), offward (OR 5.8, *P* < 0.0005, CI 3.6–9.2), and in the hall (OR 9.2, *P* < 0.0005, CI 7.0–12.2). Most of the observed day (74%) was spent in locations with low odds of light or moderate physical activity, 53% in the bedroom, 18% in the lounge, and 3% in the bathroom, while less time was spent in the therapy area (13%), the hall (9%), and offward (4%).

## 4. Discussion

There is compelling evidence that sedentary behaviour is associated with various health risks [4–10]. We found that patients in stroke rehabilitation spent as much as 74% of the “active” day sedentary. Overall patients were more active than stroke survivors in studies from acute stroke units in both Norway (77%) [14, 15] and Australia (88%) [14], but the amount of sedentary time was still high compared to a healthy population (57–57.8%) [11, 30] and for a rehabilitation setting where physical training is supposed to be a central part of the program. Bearing in mind that each additional hour spent sedentary is associated with a progressive rise in mortality risk [4], to be sedentary a total of 6.2 hours from the 8.4 hours a day participants were observed is likely to have a detrimental effect on their health. Previous studies [18, 21] have reported that patients are involved in minimal activity on evenings and weekends. Since observations in this study were carried out only during what is likely to be confirmed as the most active part of a patient's day (8:00 AM to 5:00 PM) [18] and on weekdays only, we expect that the sedentary time across all waking hours or over the whole week (including weekends) is likely to be even greater.

The results of this study showed that close to half of the observed day (44%) was spent in prolonged sedentary bouts lasting for one hour or longer without interruption. Even though rest may be needed, accumulating sedentary time in long bouts may have negative health effects. Previous research indicates that increased interruptions in sedentary bouts are associated with decreased metabolic risk [11], and this may be particularly important for people with stroke who already have a higher metabolic risk than healthy controls [31].

Another finding in this study was that physical activity levels varied during the day, between 12% and 40% of patients active at each observation. A higher proportion of patients were physically active between 9:00 AM and 12:30 PM, and this is consistent with another observational study [17]. However, the question why patients seem to be less active during the afternoon remains and may be a result of the organisation of care rather than the medical condition of the patient. It is quite reasonable that physical activity decreases during meals and both snack and dinner were served before 5:00 PM, making less time available for physical activity early in the afternoon. Opportunities to maximise activity and break up sedentary time around meals should be sought. Replacing passive transport to the dining room with supervised or assisted walking for those able to do so is one way to ensure breaks in sedentary behaviour. In our previous report examining who helps patients to be active [22], we found that patients were most active when they were with a physiotherapist or an occupational therapist. Considerably fewer patients in the current study were observed together with a therapist after 3:00 PM; however, later on the day family members were more frequently observed to be present (data not shown). Engaging the family in helping the patient to be more physically active may be a possibility to break up sedentary time and improve outcome.

Previous research [32] has demonstrated that higher amount of therapy may be attributable to a more efficient use of human resources, rather than a result of more staff. Therapists in both this and a previous study [33] were rarely observed working with more than one patient at a time, indicating further unexploited possibilities to increase therapy time and break up bouts of sedentary behaviour. Putting into practice, it could be arranged as circuit classes where therapists work closely with one patient engaged in an exercise considered to require supervision, while other patients are positioned safely practicing tasks independently and others having a short break before rotating to the next work station. A recent systematic review [34] reported that circuit class therapy is safe and time efficient, and can improve mobility in people with stroke. When provided within hospital-based rehabilitation, it was also shown to reduce length of stay. However, therapists both in our study and other settings [33] were rarely observed working with more than one patient at a time. In times of restricted resources, simultaneously engaging several patients per therapist at any one time may well be the key to increase therapy time and split up sedentary bouts, as well as integrating social aspects [18] and a more stimulating environment in the training, factors that have the potential to improve recovery. Circuit classes are not, however, likely to reduce the high rates of sedentary behaviour observed over the course of a patients' day. This would require a whole unit approach to the problem, in particular, since many patients are not independently mobile.

Interestingly, all four rehabilitation units in this study had a policy of enforced patient rest periods. Emphasizing rest on a general basis in a population already prone to spend excessive time in sedentary behaviours may not be an ideal policy, given the lack of evidence to support bed rest and the strong evidence for the negative impact of sedentary time on health [4–10]. Furthermore, the impact of fatigue after stroke and the importance of balancing rest and activity are widely acknowledged but poorly understood in this population [35]. Pathological fatigue (i.e., after stroke) has been described as a state characterised by weariness unrelated to previous exertion levels and is usually not ameliorated by rest [36]. There are hypotheses that fatigue may be associated with physical deconditioning, which is common after stroke, and that exercise, by means of reversing physical deconditioning, might reduce poststroke fatigue [35]. Extended monitoring of activity and fatigue over the course of a patients' stay may provide new insights into this problem.

In this study, we further found that physical activity varied depending on patient location. Unexpectedly, patients turned out to have higher odds of being physically active in the hall than in the therapy area. This was not in line with the only other known study in the field [21]. A possible explanation may be that the hall was sometimes used for therapy sessions in the present study, with 13% of time spent in the hall constituting involvement in light or moderate physical activity while accompanied by a therapist. In contrast to the previous study [21] the odds of being active were moreover lower in the lounge area as compared to the bedroom, indicating that there is a clear need for research investigating the environmental drivers of activity in different locations within hospitals.

When interpreting the results some limitations should be considered. As we have acknowledged previously [22], the presence of a rater may influence patient or staff behaviour and observations are intermittent so some activity may be missed. However, given that in 40% of observations patients were lying in bed, the chance that patients got out of bed and then returned between observations is low. We elected to use behavioural mapping rather than instrumented systems of measurement in this study because of the rich source of data it provides, including information about the location of patients when they are active and who was with them during the activity, which is not easily accessed by any other method. Supplementing observation with continuous activity measurement using objective activity monitors such as accelerometers or the like would be advantageous in future studies. Such a tool should be able to measure metabolic load as this would further inform definitions of sedentary behavior in people with stroke.

At present, the definition of sedentary behavior is based on MET-values in healthy individuals. People with disabilities after stroke could be expected to spend more energy than normal in many physical activities [37]; however, these activities may still be “sedentary.” In the absence of a classification system with reference values of energy expenditure in people with different disabilities after stroke, activities were grouped into categories judged by an international group of stroke rehabilitation experts to reflect the degree of physical effort required. Some activities generally considered as sedentary in healthy populations were in this study classified as physical activity. The inclusion of activities such as sitting unsupported and rolling to sit up as light physical activities may have led to an underestimation of total sedentary time.

## 5. Conclusion

Rehabilitation is a complex system of care that aims to help patients with diverse needs after stroke. It is also personnel intense and thereby very costly. Improving the efficiency of rehabilitation (better outcomes, less resources) would be welcome. The high amounts of sedentary behaviour found in this study indicate that there is potential to increase physical activity levels which may in turn improve outcome. Furthermore, the variation in activity seen across the observed day and within different areas of the rehabilitation environment suggests that it would be worthwhile to explore how hospital design and care processes could be altered to help promote activity.

## Figures and Tables

**Table 1:** Categorization of activity.

Activity category	Observed activity	Definition
(1) Nil physical activity	Lying in bed sleeping, talking, reading, eating, watching TV, or doing nothing in particular	Sedentary
(2) Minimal physical activity	Sitting supported out of bed while talking, reading, eating, or doing nothing	Sedentary
(3) Light physical activity	Rolling to sit up, sitting unsupported, or transferring with feet on floor	Physically active
(4) Moderate physical activity	Standing, walking, or climbing stairs	Physically active
